# Supplementary material for: Nursing Interventions for Patient Empowerment during Intensive Care Unit Discharge: A Systematic Review
Source: Int J Environ Res Public Health. 2021 Oct 21;18(21):11049. doi: 10.3390/ijerph182111049 (PMC8582948; doi:10.3390/ijerph182111049)
Supplement: Supplementary file 1 [file ijerph-18-11049-s001.zip › File S2. CRD42021254377.pdf]

To enable PROSPERO to focus on COVID-19 submissions, this registration record has undergone basic automated checks for eligibility and is published exactly as submitted. PROSPERO has never provided peer review, and usual checking by the PROSPERO team does not endorse content. Therefore, automatically published records should be treated as any other PROSPERO registration. Further detail is provided here.

Citation

Cecilia Cuzco, Rodrigo Torres, Pilar Delgado-Hito, Pilar Muñoz Rey, Isabel Manzanares, Yolanda Torralba, Marta Romero, M. Antonia Martínez Momblan, Pedro Castro Rebollo. Patient empowerment during intensive care unit discharge: a systematic review and meta-analysis. PROSPERO 2021 CRD42021254377 Available from: https://www.crd.york.ac.uk/prospero/display_record.php?ID=CRD42021254377

Review question

What are the nursing interventions to improve patient empowerment during ICU discharge and their effects?

Searches

We will conduct a systematic review of the literature to identify manuscripts that investigate nursing interventions in patient empowerment during ICU discharge.

We will review the Embase, Cochrane Library, CINAHL, Web of Science, and PubMed/MEDLINE databases with the followings terms:

1. For population: (ICU patients) OR (Ventilated patients) OR

2. For intervention: (empowerment patient) OR (patient education) OR (patient information)

3. For condition (ICU discharge) OR (ICU transfer) OR (ICU transition)

4. For the main outcome: (discharge experience) OR (self-care ability) OR (anxiety) OR (depression)

The terms selected will be combined using Boolean logical operators (OR, AND, NOT). Also, we will make a manual search of the references that are included in the selected articles.

All references will be analysed in the software Endnote X7.

Types of study to be included

We will include randomised clinical trials (RCTs), quasi-randomised clinical trials, and observational studies (retrospective, prospective, cross-sectional, longitudinal, case-control, and cohort). All editorials, letters to editor, review articles, systematic review, and meta-analysis, in vivo and in vitro studies will be excluded.

Condition or domain being studied

Nursing interventions for patient empowerment in the intensive care unit

Participants/population

We are studying adult patients in intensive care unit discharge.

Intervention(s), exposure(s)

Educational intervention as information, behavioural instructions and advice regarding the management of ICU discharge by verbal, written, audio or videotaped means

Comparator(s)/control

We will compare with no interventions

Main outcome(s)

1. Patient discharge experience: Measured by standardised questionnaires.

2. Relatives discharge experience: Measured by standardised questionnaires.

Measures of effect

Continuous data will be presented as the mean difference with standard deviations, or median and interquartile range. Dichotomous categorical data will be reported in the overall mean proportion (%) of the population.

Additional outcome(s)

1. Perceptions of self-care ability, measured by standardised instruments as Patient Enablement Instrument

2. Anxiety, measured by validated instruments as Hospital Anxiety and Depression Scores (HADS)

3. Depression, measured by validated instruments as Hospital Anxiety and Depression Scores (HADS)

Measures of effect

Continuous data will be presented as the mean difference with standard deviations, or median and interquartile range. Dichotomous categorical data will be reported in the overall mean proportion (%) of the population.

Data extraction (selection and coding)

We will conduct the bibliographic search and compile the identified records in Rayyan web software. We will remove any duplicated studies. Two authors (PM-IM) will work independently to extract data from included studies. All included studies will be assessed for internal validity using a checklist.

Study characteristics (design, country), baseline patient characteristics (age, demographic and anthropometric characteristics, comorbidities, hospitalization days, ICU days) and outcomes will then be extracted from the studies selected for inclusion by two reviewers (PM-IM) using a pre-designed and piloted data extraction form to avoid any errors.

Any disagreements between the reviewers will be resolved by consensus or, if necessary, through arbitration by a third reviewer (CC).

Authors may be contacted to request the provision of missing data on a case-by-case basis, considering the importance and relevance of the data which is missing.

Risk of bias (quality) assessment

The risk of bias and the quality of observational and interventional studies will be assessed using the corresponding assessment tools recommended by the National Heart, Lung, and Blood Institute (NHBLI). This assessment will be done by two investigators (CC-RT), with disagreements being resolved by arbitration by a third investigator (YT). The level of confidence in summary effect estimates will be assessed using the Grading of Recommendations Assessment, Development, and Evaluation (GRADE) approach. Publication bias will be assessed from funnel plots.

Strategy for data synthesis

A narrative synthesis will be conducted. We will report summaries of the association between the risk factor and the outcomes for each study in terms of mean differences or standardised mean differences.

If a meta-analysis is appropriate, we will estimate pooled measures of association using a random-effect meta-analysis and calculate 95% confidence intervals for each outcome. Statistical heterogeneity will be assessed by using Cochran’s Q value and the I^2 statistic from the standard ?2 test. If $I^2 > 50\%$ this will be considered to reflect significant statistical heterogeneity. When $I^2 > 50\%$, the random-effects model using the inverse variance heterogeneity method will be used. To locate the origin of the heterogeneity, sensitivity analysis, excluding one study at a time, will also be undertaken.

Analysis of subgroups or subsets

By subtype of educational intervention

Contact details for further information

Cecilia Cuzco
ccuzco@clinic.cat

Organisational affiliation of the review

Hospital Clínic de Barcelona
www.clinic.cat

Review team members and their organisational affiliations

Professor Cecilia Cuzco. Hospital Clínic de Barcelona
Professor Rodrigo Torres. Hospital Clínic de Barcelona
Dr Pilar Delgado-Hito. Universidad de Barcelona
Mrs Pilar Muñoz Rey. Hospital Germans Trias i Pujol
Mrs Isabel Manzanares. Hospital Clínic de Barcelona
Professor Yolanda Torralba. Hospital Clínic de Barcelona
Dr Marta Romero. Universidad de Barcelona
Dr M. Antonia Martínez Momblan. Universidad de Barcelona
Dr Pedro Castro Rebollo. Hospital Clínic de Barcelona

Type and method of review

Intervention, Meta-analysis, Systematic review

Anticipated or actual start date

11 June 2021

Anticipated completion date

11 August 2021

Funding sources/sponsors

Hospital Clínic de Barcelona

Conflicts of interest

Language

English

Country

Spain

Stage of review

Review Ongoing

Subject index terms status

Subject indexing assigned by CRD

Subject index terms

MeSH headings have not been applied to this record

Date of registration in PROSPERO

11 June 2021

Date of first submission

11 May 2021

Stage of review at time of this submission

The review has not started

Stage	Started	Completed
Preliminary searches	No	No
Piloting of the study selection process	No	No
Formal screening of search results against eligibility criteria	No	No
Data extraction	No	No
Risk of bias (quality) assessment	No	No
Data analysis	No	No

The record owner confirms that the information they have supplied for this submission is accurate and complete and they understand that deliberate provision of inaccurate information or omission of data may be construed as scientific misconduct.
The record owner confirms that they will update the status of the review when it is completed and will add publication details in due course.

Versions

11 June 2021
11 June 2021
